# Patient-reported outcomes and safety in patients undergoing synovial biopsy: comparison of ultrasound-guided needle biopsy, ultrasound-guided portal and forceps and arthroscopic-guided synovial biopsy techniques in five centres across Europe

**DOI:** 10.1136/rmdopen-2018-000799

**Published:** 2018-10-26

**Authors:** Søren Andreas Just, Frances Humby, Hanne Lindegaard, Laurent Meric de Bellefon, Patrick Durez, Elsa Vieira-Sousa, Rui Teixeira, Maria Stoenoiu, Jens Werlinrud, Sofie Rosmark, Pia Veldt Larsen, Arthur Pratt, Ernest Choy, Nagui Gendi, Maya H Buch, Christopher J Edwards, Peter C Taylor, Iain B McInnes, João Eurico Fonseca, Costantino Pitzalis, Andrew Filer

**Affiliations:** 1 Department of Rheumatology, Odense University Hospital, Odense, Denmark; 2 Experimental Medicine and Rheumatology, William Harvey Research Institute, Queen Mary University of London, London, UK; 3 Department of Rheumatology, Saint-Pierre University Hospital, Brussels, Belgium; 4 Rhumatologie, Cliniques Universitaires Saint-Luc, Institut de Recherche Expérimentale et Clinique (IREC), Bruxelles, Belgium; 5 Instituto de Medicina Molecular, Faculdade de Medicina, Universidade de Lisboa, Lisbon, Portugal; 6 Rheumatology Department, Hospital de Santa Maria, CHLN, Lisbon Academic Medical Centre, Lisbon, Portugal; 7 Department of Orthopedics, Odense University Hospital, Odense, Denmark; 8 Epidemiology and Biostatistics, Department of Public Health, University of Southern Denmark, Odense, Denmark; 9 Institute of Cellular Medicine, Newcastle University, Newcastle, UK; 10 Newcastle NIHR Biomedical Research Centre, Newcastle upon Tyne Hospitals NHS Foundation Trust and Newcastle University, Newcastle, UK; 11 CREATE Centre, Section of Rheumatology, Division of Infection and Immunity, Cardiff University School of Medicine, Cardiff, UK; 12 Basildon University Hospital, Basildon, UK; 13 Leeds Institute of Rheumatic and Musculoskeletal Medicine, University of Leeds, Leeds, UK; 14 NIHR Leeds Musculoskeletal Biomedical Research Centre, Leeds, UK; 15 Southampton MSK Research Unit, NIHR Clinical Research Facility, University Hospital Southampton, Southampton, UK; 16 Botnar Research Centre, NDORMS, Oxford University, Oxford, UK; 17 Institute of Infection, Immunity and Inflammation, University of Glasgow, Glasgow, UK; 18 Institute of Inflammation and Ageing, College of Medical and Dental Sciences, University of Birmingham, Edgbaston, UK

**Keywords:** patient perspective, arthritis, rheumatoid arthritis, early rheumatoid arthritis, ultrasonography

## Abstract

**Background:**

We present a European multicenter study, comparing safety data and patient-reported outcomes (PRO) from patients undergoing synovial biopsy using ultrasound-guided needle biopsy (US-NB), ultrasound-guided portal and forceps (US-P&F) or arthroscopic-guided (AG) procedures.

**Objectives:**

To describe safety and PRO data on joint indices of pain, stiffness and swelling before and after biopsy, procedural discomfort, joint status compared with before biopsy and willingness to undergo a second biopsy for each technique and compare the three techniques. To evaluate the impact on PRO and safety data of corticosteroid therapy as part of the biopsy procedure and sequential biopsy procedures.

**Methods:**

Data were collected on the day of biopsy and 7–14 days postprocedure. Joint pain, swelling and stiffness indices were recorded as 0–100  mm Visual Analogue Scale; qualitative outcome variables on five-point Likert scales. Groups were compared with linear regression, adjusting for disease activity, corticosteroid therapy and prebiopsy PRO value and accounting for repeated measurements.

**Results:**

A total of 524 synovial biopsy procedures were documented (402 US-NB, 65 US-P&F and 57 AGSB). There were eight adverse events (1.5%) with no difference between biopsy methods (p=0.55). All PROs were improved 2  weeks postprocedure, and there were no differences in postbiopsy change in PROs between biopsy methods. Corticosteroid administration, whether intramuscular (n=62) or intra-articular (n=38), did not result in more adverse events (p=0.81) and was associated with reduction in postbiopsy swelling (p<0.01). Sequential biopsy procedures (n=103 patients) did not result in more adverse events (p=0.61) or worsening in PRO data.

**Conclusion:**

Overall, our results do not suggest a significant difference in safety or patient tolerability between US-NB, US-P&F and AGSB sampling. Further, corticosteroid therapy as part of the biopsy procedure and sequential biopsies is safe and well tolerated in patients.

**Key messages**

**What is already known about this subject?**

- Arthroscopic-guided synovial biopsy, ultrasound-guided needle biopsy and ultrasound-guided portal and forceps methods has been shown to be a safe and well-tolerated procedures, although without comparison of patient-reported outcomes and safety between the methods.

**What does this study add?**

- This is the largest comparative study evaluating the tolerability and safety of ultrasound-guided and arthroscopic biopsy techniques and we found no significant difference between ultrasound-guided needle biopsy, ultrasound-guided portal and forceps or the arthroscopic-guided synovial biopsy methods.
- Further, corticosteroid therapy as part of the biopsy procedure and sequential biopsies is safe and well tolerated in patients.

**How might this impact on clinical practice?**

- From a safety and patient perspective, all three synovial biopsy methods can be used in both trials and clinical practice.

## Introduction

Synovial tissue analysis could provide a step change towards personalising diagnosis, disease stratification and treatment selection of patients with inflammatory arthritis.^1 2^Arthroscopic-guided synovial biopsy (AGSB) is to date regarded as the ‘gold standard’ for synovial tissue acquisition due to extensive validation examining synovial biopsy performance and patient tolerability and safety.^3 4^ However, the technical skills and equipment required generally restrict the use of arthroscopy for research purposes to targeting large joints and within academic centres in rheumatology practice.^2 4–6^ By contrast, minimally invasive ultrasound-guided needle biopsy (US-NB) or portal and forceps (US-P&F) are becoming widely adopted.^7–9^ These approaches permit access to synovial tissue in a wider range of joints, are technically simple, relatively inexpensive to perform and have a generally good safety record.^5 7 10 11^ However, the wide scale adoption of US-guided techniques in clinical trials and in routine care requires further studies evaluating patient safety and tolerability of the techniques, particularly compared with the gold standard of arthroscopy.^4^

The aim of this multicenter retrospective cohort study of patients with inflammatory arthritis undergoing US-NB, US-P&F and AGSB was to evaluate whether there were significant differences in safety and tolerability between the techniques.

## Patients and methods

### Participating centers

The study protocol was presented at the European Synovitis Study Group European League Against Rheumatism (EULAR) meeting in June 2017. Centres with consecutive cohorts of patients with inflammatory arthritis undergoing US-NB or US-P&F and AGSB procedures with patient-reported outcome (PRO) data and safety data were invited to participate in the study. Academic rheumatology centres with suitable cohorts of patients included in the study were: Barts and the London School of Medicine and Dentistry (UK) (data from the multicenter studies Stratification of Biologic Therapies for Rheumatoid Arthritis by Pathobiology (STRAP) and Response—Resistance to Rituximab vs Tocilizumab in Rheumatoid Arthritis (R4RA) trials), Odense University Hospital (Denmark) (data from the SynRA study), Birmingham (UK) (data from the Birmingham Early Arthritis Cohort (BEACON)), Brussels (Belgium) and Lisbon Academic Medical Centre (data from mini-arthroscopy cohort, Portugal).

### Biopsy procedures

#### US-guided needle biopsy

**Figure 1:**
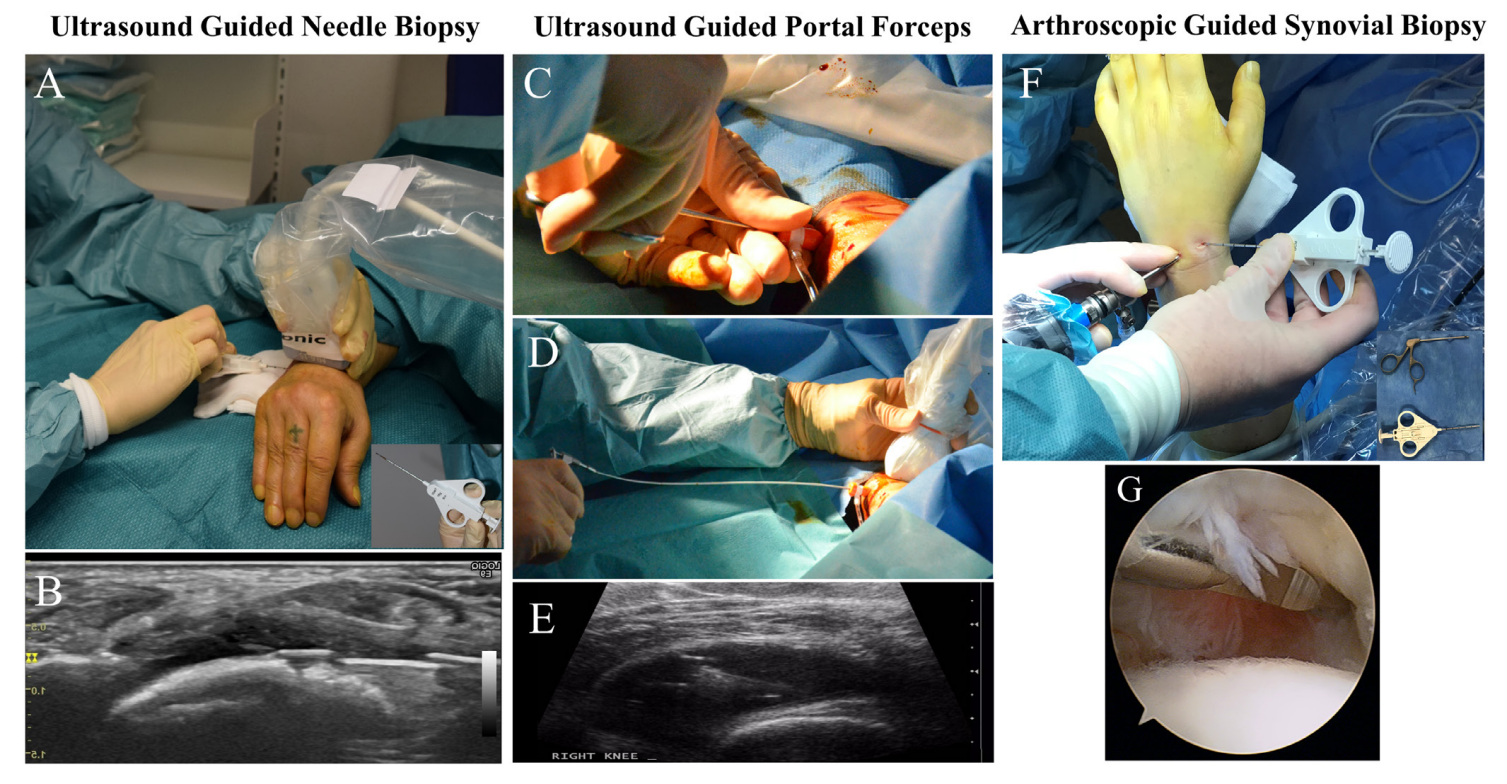
(A) The ultrasound-guided needle biopsy (US-NB) procedure of wrist, in outpatient clinic using local anaesthesia. In the lower right corner example of biopsy needle. (B) Image of ultrasound guidance during US-NB procedure. (C) and (D) Portal and forceps technique: using rigid and flexible forceps through a disposable portal under local anaesthesia, (E) Synovial sampling within the knee joint under ultrasound control. (F) Arthroscopy of wrist, in operating theatre, patient under general anaesthesia. In lower corner examples of biopsy equipment. (G) Video guidance used to visualise synovium during arthroscopy.

US-NB procedures (figure 1A) were performed in a clean procedure room or operating theatre, on both large joints and small joints, as previously described.^7^ Briefly, local anaesthetic was injected into the soft tissue up to the joint capsule and into the joint space and a Quick-Core biopsy needle (16/14-gauge; Cook Medical) was then guided by ultrasound and placed within the joint capsule (figure 1B) to retrieve synovium.

#### US-guided portal and forceps

US-P&F procedures (figure 1C–E) were performed in a clean procedure room on large joints (knee, ankle and wrist).^8 9^ Briefly, local anaesthetic was injected into the soft tissue up to the joint capsule and into the joint space. A single 7 Fr disposable portal was placed using Seldinger technique under ultrasound guidance, within the joint capsule. Hereafter, synovial biopsies were obtained, through the portal, using rigid 2.2 mm (Hartmann Herzfeld, Medicon, Germany) and flexible 2.2 mm (Tontarra, Germany) forceps (figure 1C–E).

#### Arthroscopic-guided synovial biopsy

AGSB procedures (figure 1F) were performed in an operating theatre setting or a clean procedure room, on both small and large joints, as previously described.^5 12 13^ Arthroscopy was performed using local, regional or general anaesthesia.

Briefly, for the wrist joint, patients underwent general anaesthesia and up to 5 kg of traction across the wrist. Two standard arthroscopic portals were inserted into the radiocarpal joint, one for arthroscopic visualisation and the other for instrumentation. Synovial biopsies were obtained under direct visual inspection (figure 1G), using a Quick-Core biopsy needle (16/14-gauge; Cook Medical) (figure 1F) a standard wrist arthroscopy punch was also used (figure 1F, lower right corner insert). For the knee joint, standardised procedures for synovial tissue collection in clinical trials, under local anaesthesia, were followed as previously described.^13^ Arthroscopies were performed with (‘wet’) and without (‘dry’) flow of saline through the joint during the procedure.

### Patients

Patient characteristics including age, gender, diagnosis, disease duration, joint biopsied, C-reactive protein (CRP), erythrocyte sedimentation rate (ESR) and rheumatological medication (conventional synthetic (cs) disease-modifying antirheumatic drugs (csDMARD), biological DMARDs (bDMARDs), corticosteroid), disease activity score in 28 joints with CRP or ESR (DAS28CRP or DAS28ESR), use of intramuscular (IM) or intra-articular (IA) corticosteroids immediately following biopsy were collected.

### PRO data

Prebiopsy (day of biopsy) and postbiopsy (7–14 days postprocedure) pain, stiffness and swelling (Visual Analogue Scale (VAS) from 0 to 100 mm) were collected for each biopsy procedure. Further, PROs if available, on the degree of discomfort during the procedure, prebiopsy and postbiopsy evaluation of worsening/improvement of joint symptoms and the willingness of the patient to undergo another biopsy were also collected using five-point Likert scales.

### Safety data

All registered adverse events (no time restriction) between time of biopsy to November 2017 were collected, including, but not limited to, data on presyncope, syncope, tenosynovitis, joint infection, deep vein thrombosis, nerve or tendon damage or haemarthrosis.

### Statistical analysis

Categorical variables on patient characteristics were presented as numbers and percentages, and continuous variables as means with SD. Between-group PRO data comparisons were performed using both unadjusted and adjusted linear regression, adjusting for biopsy method, disease activity, IM or IA corticosteroid use during biopsy procedure and the prebiopsy PRO value.^14^ Robust cluster estimation was used in the linear regressions to account for minor deviations from the model assumptions on normality and to account for repeated measurements of some patients. Differences in answers between biopsy methods in the questions measured on Likert scales were analysed by multinomial logistic regression using robust cluster estimation and adjusting for disease activity and IM or IA corticosteroid used during biopsy procedure. In the analysis of discomfort during procedure, AGSB procedures performed in general anaesthesia were excluded. Tests for interaction between biopsy technique and use of peri-biopsy IM or IA corticosteroid were conducted in relation to the PRO outcomes on discomfort and rebiopsy. Comparison of PRO data in the cohort of patients with repeated biopsies was performed using linear regression with robust cluster estimation, both unadjusted and adjusting for disease activity, corticosteroid use during biopsy procedure and in post-PRO values, the prebiopsy PRO data value. P values <0.05 were taken as statistically significant. Data were analysed on Stata V.15.

## Results

### Patients and procedures

Data from a total of 524 synovial biopsy procedures were included from 421 patients. Of these, 43% (228/524) of the procedures were performed as part of the multicentre R4RA (14 centres) and STRAP (nine centres) trials led by Barts and the London, while the remaining procedures were included from Brussels, n=122 (23%), Odense, n=98 (19%), Birmingham, n=56 (11%) and Lisbon, n=20 (4%) centres. A total of 318 patients (75.5%) underwent one synovial biopsy and 103 (24.5%) a repeat biopsy. Overall, 77% (402/524) of procedures were performed using US-NB, 12% (65/524) were performed using US-P&F and 11% (57/524) were performed using AGSB. For details on distribution of biopsy methods between centres, please see online supplementary table 1A and B. In the arthroscopy group, 77% (44/57) were performed using local anaesthesia and 23% (13/57) under general anaesthesia. In the US-NB group, biopsies were primarily from the wrist (69%) and, as the only method, also from small joints as metacarpophalangeal (MCP) (18%) and metatarsophalangeal (MTP) (1%) joints. In the US-P&F and arthroscopy groups, the primary joint biopsied was the knee (86% and 68%, respectively). Characteristics of patients at the time of biopsy are shown in table 1

10.1136/rmdopen-2018-000799.supp1 Supplementary data

**Table 1:** Patient characteristics

	US-NB	US-P&F	AGSB	Total	P values	Missing (%)
Biopsy procedures, n (%)	402 (76.7)	65 (12.4)	57 (10.9)	524 (100.0)		
Age, mean (SD)	55.93 (13.79)	52.43 (12.07)	52.30 (17.00)	55.12 (14.00)	0.05	1.9
Gender, n (%)						
Female, n (%)	285 (72.5)	35 (53.8)	38 (70.4)	358 (69.9)	0.01	2.3
Diagnosis, n (%)						
Rheumatoid arthritis, n (%)	360 (89.6)	42 (64.6)	27 (47.4)	429 (81.9)		
Undifferentiated arthritis, n (%)	20 (5.0)	8 (12.3)	2 (3.5)	30 (5.7)		
Spondyloarthritis, n (%)	7 (1.7)	0 (0.0)	2 (3.5)	9 (1.7)		
Psoriatic arthritis, n (%)	7 (1.7)	9 (13.8)	2 (3.5)	18 (3.4)		
Degenerative, n (%)	0 (0.0)	0 (0.0)	13 (22.8)	13 (2.5)		
Other, n (%)	8 (2.0)	6 (9.2)	11 (19.3)	25 (4.8)	<0.01	0
Disease duration (years)	8.21 (9.74)	0.53 (1.35)	9.09 (9.40)	7.25 (9.42)	<0.01	8.5
Time from diagnosis to biopsy						
Under 1 month, n (%)	53 (13.2)	40 (61.5)	1 (1.8)	94 (17.9)		
One to 12 months, n (%)	42 (10.4)	15 (23.1)	11 (19.3)	68 (13.0)		
Over 1 year, n (%)	307 (76.4)	10 (15.4)	45 (78.9)	362 (69.1)	<0.01	0
Joint biopsied						
Wrist, n (%)	277 (69.3)	1 (1.5)	18 (31.6)	296 (56.7)		
Knee, n (%)	34 (8.5)	56 (86.2)	39 (68.4)	129 (24.7)		
MCP, n (%)	74 (18.5)	0 (0.0)	0 (0.0)	74 (14.2)		
Ankle, n (%)	2 (0.5)	8 (12.3)	0 (0.0)	10 (1.9)		
Elbow, n (%)	8 (2.0)	0 (0.0)	0 (0.0)	8 (1.5)		
MTP, n (%)	5 (1.3)	0 (0.0)	0 (0.0)	5 (1.0)	<0.01	0.4
Disease activity score (DAS28-CRP/ESR), n (%)						
EULAR remission	28 (10.4)	4 (6.3)	2 (5.7)	34 (9.3)		
EULAR low disease activity	14 (5.2)	7 (11.1)	7 (20.0)	28 (7.6)		
EULAR moderate disease activity	98 (36.4)	31 (49.2)	18 (51.4)	147 (40.1)		
EULAR high disease activity	129 (48.0)	21 (33.3)	8 (22.9)	158 (43.1)	<0.01	29
Treatment						
Naive	131 (35.2)	58 (89.2)	25 (51.0)	214 (44.0)	<0.01	7.2
csDMARDs	218 (58.6)	7 (10.8)	23 (46.9)	248 (51.0)	<0.01	7.2
Oral corticosteroid	64 (15.9)	2 (3.1)	13 (22.8)	79 (15.1)	<0.01	7.2
bDMARDs	53 (14.2)	1 (1.5)	1 (2.0)	55 (11.3)	<0.01	7.2
IM or IA corticosteroid at biopsy procedure						
IM corticosteroid at biopsy, n (%)	55 (13.7)	3 (4.6)	4 (7.0)	62 (11.8)		
IA corticosteroid at biopsy, n (%)	26 (6.5)	0 (0.0)	12 (21.1)	38 (7.3)	<0.01	0

AGSB, arthroscopic-guided synovial biopsy; CRP, C-reactive protein; DMARD, disease-modifying antirheumatic drugs; ESR, erythrocyte sedimentation rate; EULAR, European League Against Rheumatism; IA, intra-articular; IM, intramuscular; MCP, metacarpophalangeal; MTP, metatarsophalangeal; US-NB; ultrasound-guided needle biopsy; US-P&F, ultrasound-guided portal and forceps.

When patients were categorised according to biopsy technique, significant differences in gender, diagnosis, disease duration, time from diagnosis to biopsy, joint biopsied and EULAR disease activity criteria between groups were demonstrated, reflecting a diversity in the patients with inflammatory arthritis recruited at each centre. In addition, significant differences in rheumatological medication were found between biopsy groups: 44% (214/524) of patients were treatment naive with the greatest proportion in the US-P&F group (89.2% (58/65), p<0.01). In total, 51%, (248/524) of patients were being treated with one or more csDMARD. The prevalence of bDMARDs was highest in the US-NB group (14% (53/402), p<0.01), while use of steroid during biopsy procedure (IM or IA) was lowest in the US-P&F group (3% (3/63), p<0.01). Detailed information on csDMARD and bDMARD therapy is shown in online supplementary table 2.

### Tolerability of biopsy procedures

In order to determine whether there were significant differences in tolerability of the procedures between the three biopsy techniques, we next evaluated differences in pain, stiffness and swelling before and after the biopsy procedure. Although we found significantly higher prebiopsy levels of pain and swelling within the arthroscopic group (p<0.01), we found no significant differences in postbiopsy pain, swelling and stiffness levels. Further, we found that the changes in pain, stiffness and swelling from baseline were not significantly different between biopsy techniques (online supplementary table 3).

**Figure 2:**
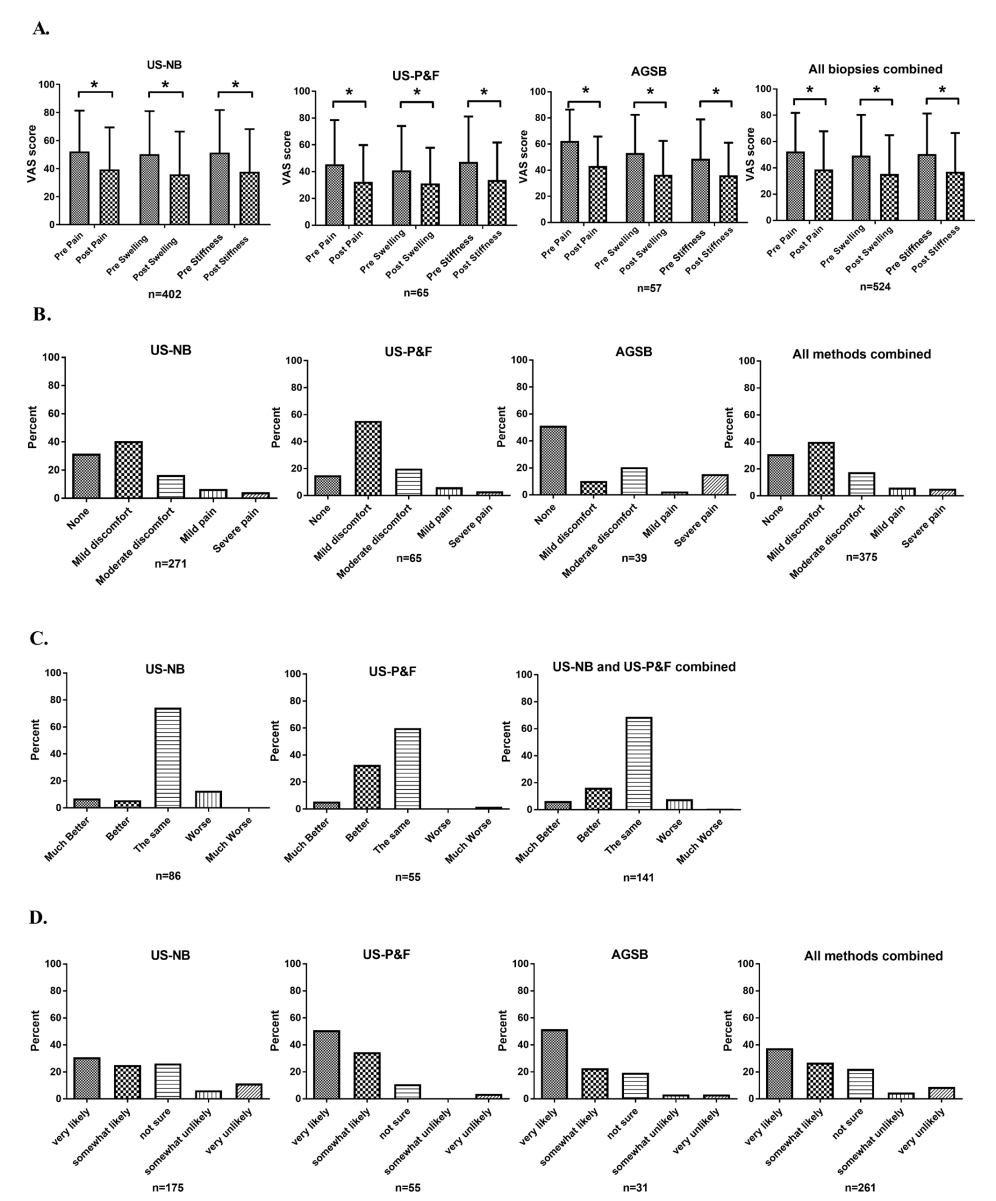
(A) Graphs of patient reported data on Visual Analogue Scale for (a) ultrasound-guided needle biopsy (US-NB), (b) ultrasound-guided portal and forceps (US-P&F) and (c) arthroscopic-guided synovial biopsy (AGSB) and (d) all methods combined are presented. *P<0.05. (B) Answer to question on discomfort during procedure. The AGSB procedures using general anaesthesia not included. (C) Answer to question on how the joint feels during compared with before biopsy. Arthroscopy not applicable due to small amount of data. (D) Answer to question on whether the patient would participate in another biopsy.

For all patients biopsied, irrespective of method and for each method individually, we found that pain, swelling and stiffness all decreased significantly after the procedure (figure 2A). These results indicate that all biopsy techniques evaluated were generally well tolerated, often with significant reduction in pain, swelling and stiffness of joints following biopsy.

Among patients undergoing procedures under local anaesthesia, we found that a high proportion of patients undergoing each biopsy technique experienced absent or only mild discomfort (US-NB 72% (196/271), US-P&F 71% (46/65), AGSB 62% (24/39)), we found no significant difference in outcomes in the adjusted analysis between methods (p=0.72) (figure 2B).

In the patients undergoing US-NB and US-P&F, we were also able to evaluate answers to the question ‘How does joint feel compared with before biopsy’ (figure 2C). In both the US-NB and US-P&F group, 87% (75/86) and 99% (54/55), respectively, of the patients answered that the joint felt the same or better. When comparing the answers in adjusted analysis, the US-P&F group scored better compared with the US-NB group (p<0.01).

Finally, we evaluated differences regarding willingness to undergo a repeat biopsy (figure 2D). In all groups, the majority of patients were somewhat or very likely to agree to another biopsy (US-NB 57% (98/175), US-P&F 85% (47/55), AGSB 64% (23/31)). Adjusted analysis demonstrated that patients undergoing both US-P&F and AGSB were more willing than those undergoing US-NB to undergo a repeat procedure (US-P&F vs US-NB: p<0.01 and AGSB vs US-NB: p<0.01), while there was no significant difference between US-P&F and AGSB (p=0.85).

### Outcomes in patients undergoing repeated biopsy procedures

In the 103 patients who had undergone two biopsy procedures, we evaluated differences in VAS scores for preprocedure and postprocedure pain, stiffness and swelling and differences in willingness to undergo a repeat biopsy. Of the 103 patients, the majority (84.5%, (87/103)) had undergone US-NB and 11% (11/103) US-P&F and 5% (5/103) AGSB (table 2).

**Table 2:** Repeated biopsy procedures

	Baseline	Repeat biopsy	P values	P values*	Missing (%)
n	103	103			
Months between biopsies†﻿		5.37 (5.50)			
Biopsy method					
US-NB, n (%)	87 (84.5)	88 (85.4)			
US-P&F, n (%)	11 (10.7)	11 (10.7)			
AGSB, n (%)	5 (4.9)	4 (3.9)	0.94		
Diagnosis					
RA, n (%)	100 (97.1)	100 (97.1)			
UA, n (%)	1 (1.0)	1 (1.0)			
PsA, n (%)	2 (1.9)	2 (1.9)	1.00		
Joint biopsied					
Elbow, n (%)	2 (1.9)	2 (1.9)			
Wrist, n (%)	70 (68.0)	70 (68.0)			
MCP, n (%)	8 (7.8)	8 (7.8)			
Knee, n (%)	20 (19.4)	20 (19.4)			
Ankle, n (%)	2 (1.9)	2 (1.9)			
MTP, n (%)	1 (1.0)	1 (1.0)	1.00		0.4
EULAR disease activity					
Remission, n (%)	0 (0.0)	28 (43.8)			
Low activity, n (%)	3 (3.4)	4 (6.3)			
Moderate activity, n (%)	42 (47.7)	21 (32.8)			
High activity, n (%)	43 (48.9)	11 (17.2)	<0.01		29.9
Patient-reported outcome measures					
Pre-pain, mean (SD)	54.96 (28.01)	32.90 (26.01)	<0.01	<0.01	2.5
Post-pain, mean (SD)	39.30 (29.29)	27.05 (24.12)	<0.01	0.6	7.5
Pre-swelling, mean (SD)	50.46 (29.56)	28.70 (25.32)	<0.01	<0.01	5.3
Post-swelling, mean (SD)	34.94 (30.55)	20.14 (22.79)	<0.01	<0.01	9.5
Pre-stiffness, mean (SD)	57.16 (27.62)	31.11 (24.27)	<0.01	<0.01	5.3
Post-stiffness, mean (SD)	35.60 (29.55)	22.54 (23.84)	<0.01	0.52	9.3
Delta pain, mean (SD)	−15.23 (29.93)	−5.53 (21.13)	0.01	0.62	7.6
Delta swelling, mean (SD)	−15.04 (31.87)	−7.67 (26.41)	0.06	0.70	9.9
Delta stiffness, mean (SD)	−21.30 (32.70)	−7.90 (21.58)	<0.01	0.52	9.7
Participate in another biopsy?					
Very unlikely, n (%)	6 (10.5)	5 (8.6)			
Somewhat unlikely, n (%)	2 (3.5)	3 (5.2)			
Not sure, n (%)	8 (14.0)	16 (27.6)			
Somewhat likely, n (%)	8 (14.0)	8 (13.8)			
Very likely, n (%)	33 (57.9)	26 (44.8)	0.25	0.51	50.2

*P values adjusted for baseline or follow-up biopsy, disease activity, intra-articular or intramuscular corticosteroid injection during biopsy. P-values on post and delta values were also adjusted for prevalue.

†Mean for all in group.

EULAR, European League Against Rheumatism; GSB, arthroscopic-guided synovial biopsy; MCP, metacarpophalangeal; MTP, metatarsophalangeal; PsA, psoriatic arthritis; RA, rheumatoid arthritis; UA, undifferentiated arthritis; US-NB, ultrasound-guided needle biopsy; US-P&F, ultrasound-guided portal and forceps.

The majority of repeat biopsies were performed in patients with rheumatoid arthritis (97.1% (100/103)) who were biopsied from the wrist (68% (70/103)). Disease activity was significantly higher on first biopsy compared with second (96.6% moderate or high disease activity at biopsy 1 vs 44.5% at second biopsy, p<0.01), with 44% in remission at the second biopsy with 0% at the first biopsy (table 2).

Comparing PRO data between the first and second biopsy, adjusting for potential confounders, we demonstrated lower pre-pain (p<0.01), pre-stiffness (p<0.01) and pre-swelling and post-swelling (both p<0.01) in patients undergoing a second biopsy, while other PRO data were not significantly different. No statistically significant differences were found in delta PRO values between first and second biopsy (table 2).

When evaluating differences in whether patients were willing to undergo a repeat biopsy between first and second biopsy, we saw no differences in whether they were somewhat likely or very likely to undergo a repeat biopsy (first biopsy 72% (41/57), second biopsy 59% (34/58)). Notably, there was no significant decline in willingness between first and second biopsy (p=0.51). Further, there were no significant differences in adjusted analyses of VAS data (pain, stiffness, swelling or delta values) between biopsy techniques in patients undergoing repeated biopsies (data not shown).

### The effect of peri-biopsy corticosteroid therapy on PRO data

In order to determine whether the administration of peri-biopsy corticosteroids influenced patient tolerability, we evaluated whether significant differences in pre-pain and post-pain, stiffness and swelling for each biopsy technique differed according to administration of IM or IA corticosteroid.

First, we evaluated all procedures (n=424) where treatment with peri-biopsy corticosteroid was not used and in adjusted analysis found significant decreases in pain, stiffness and swelling after biopsy in the US-NB (n=321), US-P&F (n=62) and AGSB procedures (n=16) (table 3).

**Table 3:** Effect of peri-biopsy corticosteroid therapy

Prebiopsy	Postbiopsy	P values*	P values^†^
**Without IM or IA steroid**				
US-NB (n=321)				
Pain (mean (SD))	53.6 (28.9)	41.4 (30.4)	<0.001	<0.001
Stiffness (mean (SD))	51.9 (30.4)	39.4 (30.8)	<0.001	<0.001
Swelling (mean (SD))	50.9 (30.9)	38.2 (30.1)	<0.001	<0.001
US-P&F (n=62)				
Pain (mean (SD))	43.7 (32.5)	31.6 (27.2)	0.003	0.005
Stiffness (mean (SD))	45.5 (33.7)	33.4 (27.6)	0.003	0.006
Swelling (mean (SD))	39.3 (32.7)	30.7 (26.4)	0.021	0.029
AGSB (n=41)				
Pain (mean (SD))	62.3 (23.5)	49.3 (22.4)	0.017	0.001
Stiffness (mean (SD))	45.4 (31.5)	38 (26.6)	0.296	0.001
Swelling (mean (SD))	49.6 (30.9)	41.7 (28.2)	0.198	<0.001
**With IM or IA steroid**				
US-NB (n=81)				
Pain (mean (SD))	46.3 (29.4)	31.8 (26.9)	0.001	<0.001
Stiffness (mean (SD))	49.8 (30.1)	31.2 (28.2)	<0.001	0.001
Swelling (mean (SD))	47.8 (29.9)	27.3 (27.7)	<0.001	<0.001
US-P&F (n=3)				
Pain (mean (SD))	84.7 (7.2)	49.3 (33.5)	0.173	0.279
Stiffness (mean (SD))	83.7 (8.1)	40.7 (42.4)	0.192	0.304
Swelling (mean (SD))	76 (21.3)	42 (36)	0.189	0.300
AGSB (n=16)				
Pain (mean (SD))	62.9 (26)	28.9 (16.1)	<0.001	<0.001
Stiffness (mean (SD))	57.5 (25.2)	31.8 (20.4)	<0.001	<0.001
Swelling (mean (SD))	61.9 (23.0)	24.2 (14.2)	<0.001	<0.001

*P value by unadjusted cluster linear regression.

†Here adjusted for disease activity and pre-PRO value.

AGSB, arthroscopic-guided synovial biopsy; IA, intra-articular; IM, intramuscular; US-NB, ultrasound-guided needle biopsy; US-P&F, ultrasound-guided portal and forceps.

In the cohort of patients treated with peri-biopsy corticosteroids (n=100) (US-NB (n=81), US-P&F (n=3), AGSB (n=16)), we demonstrated a significant decrease in all three PRO parameters for US-NB and both pain and stiffness for arthroscopic biopsy (table 3). In the US-P&F group, there was insufficient data (only three procedures) to demonstrate a possible decrease in the three PRO parameters.

When all procedures were combined and adjusted for biopsy method, disease activity and pre-PRO value, the use of corticosteroid significantly reduced post-swelling VAS data (p<0.01), but not postprocedure pain (p=0.15) or stiffness (p=0.13).

We found no interaction between biopsy technique and use of peri-biopsy IM or IA corticosteroid in relation to the discomfort (p=0.33) or rebiopsy questions (p=0.95). Analysis was not possible for ‘How joints feel compared with before biopsy’ in the arthroscopy group due to low numbers.

### Safety data

We next collected data on all adverse events per biopsy technique and evaluated whether significant differences in rates of complications existed between procedures. Safety data was available on all procedures. Biopsy procedures were performed in the time interval between September 2012 and November 2017, with follow-up data available for a mean of 16 months (US-NB 15 months, US-P&F 12 months, AGSB 23 months).

Overall, there were n=8 (1.5%) adverse events. Although all complications were reported within the US-guided procedure group, overall there was no difference in number of adverse events between the biopsy methods (p=0.55) (table 4). The most common complication reported was postprocedure sensory disturbance (n=4), all within US-NB procedures.

**Table 4:** Safety data

Safety	US-NB	US-P&F	AGSB	Total	P values
Biopsy procedures, n (%)	402 (76.7)	65 (12.4)	57 (10.9)	524 (100.0)	
Syncope or presyncope, n (%)	2 (0.8)	0 (0.0)	0 (0.0)	2 (0.4)	
Tenosynovitis	1 (0.3)	0 (0.0)	0 (0.0)	1 (0.2)	
Neurological disturbance*, n (%)	4 (1.0)	0 (0.0)	0 (0.0)	4 (1.0)	
Tendon damage, n (%)	0 (0.0)	0 (0.0)	0 (0.0)	0 (0.0)	
Haemarthrosis, n (%)	1 (0.3)	0 (0.0)	0 (0.0)	1 (0.2)	
Deep vein thrombosis	0 (0.0)	0 (0.0)	0 (0.0)	0 (0.0)	
Joint infection	0 (0.0)	0 (0.0)	0 (0.0)	0 (0.0)	
Total adverse events	8	0	0	8	0.55†

*Sensory impairment following biopsy, no motor involvement.

†Test for difference in total number of adverse events in each group, by Fishers exact.

AGSB, arthroscopic-guided synovial biopsy; US-NB, ultrasound-guided needle biopsy; US-P&F, ultrasound-guided portal and forceps.

Further, in the 100 procedures in which IM (n=62) or IA (n=38) corticosteroid therapy was administered we also evaluated the effect on adverse events. We found no higher risk of adverse events in the group receiving corticosteroid (p=0.81). Neither was there a higher risk in patients receiving bDMARDs at the time of biopsy (n=55, p=0.63). In the patients undergoing two biopsies there was no higher risk of adverse events when undergoing the second biopsy procedure compared with first (p=0.61).

## Discussion

We herein present a multicentre study across European academic rheumatology centres evaluating PRO and safety data from 524 synovial biopsy procedures where either US-NB, US-P&F or AGSB techniques where used. Our results demonstrate a number of important findings. First, all three synovial biopsy techniques were safe and well tolerated with no significant differences demonstrated between techniques in PROs or adverse events demonstrated. Second, patients are broadly willing to undergo repeat ultrasound-guided procedures, and this willingness does not decrease when repeats occur. Third, our results suggest that peri-biopsy administration of IM or IA corticosteroid is safe and can improve tolerability of the procedure. Finally, our results suggest no new safety or tolerability issues in a large cohort of patients undergoing sequential synovial biopsy using any of the three techniques.

To our knowledge, this is the largest comparative study evaluating the tolerability and safety of US-guided and arthroscopic biopsy techniques. Such an evaluation is important as the application of US-guided techniques to sample synovial tissue has become increasingly common, driven by a minimally invasive approach with no requirement for operating theatre time and studies supporting the quality of synovial tissue obtained using these procedures.^4 7 15^ However, the previously held perception that minimally invasive US-guided procedures are better tolerated than arthroscopy is not supported by our data, suggesting that patients are able to tolerate all procedures with minimal discomfort or postprocedure pain.^4^ It is important to note that for all three biopsy techniques, the majority of patients would agree to a subsequent biopsy. Furthermore, despite a significant fall in disease activity measures in patients prior to undergoing a second biopsy, with a consequent reduction in the inflamed synovial tissue mass, available to sample, there were no significant increases in PROs including postprocedure pain, swelling or stiffness.

Further adding to the methods tolerability, in patients not receiving IM or IA steroid treatment, there was an overall improvement in postbiopsy pain, swelling and stiffness (table 3). This effect could be driven by other factors such as start or change of treatment, patient reassurance, good experience with procedure and treatment staff and the effect of more attention given to patients in clinical studies. The focus of this study was that there was no worsening in PRO data.

When considering the integration of synovial biopsy into clinical trials recruiting patients with highly active disease, peri-biopsy administration of corticosteroids is important for subsequent swift and effective control of synovitis. However, it is uncertain whether this is associated with an increased risk of adverse events. Our data suggests that clinicians can safely administer corticosteroids following synovial sampling with any of the three techniques examined and that corticosteroids reduced patient reported postprocedure swelling. It is also important to note that our results do not suggest an increased risk of adverse events in patients treated with biologics undergoing synovial biopsy.

The results on safety presented here are in line with previous studies. AGSB has been shown to be a safe and well-tolerated procedure with a low rate of complications.^3 5 16^ Both US-NB and US-P&F methods have previously been found reliable, safe and tolerable in both small and large joints in smaller cohorts.^7–9^

The comparative examination in this study of 524 synovial biopsy procedures remains the largest reported cohort to date, and the inclusion of patients with both early and established disease and with a wide variation in disease activity suggests the results may be generalisable. The study has limitations including the heterogenous characteristics of the patient cohorts, possible difference in number of retrieved biopsies per procedure, the relatively small number of AGSB procedures available for comparison, the number of contributing biopsy centres and the retrospective nature of the study. Nor were we able to take into account the ‘operator’ effect on biopsy outcomes and the procedure allocation. The tolerability and the PRO measures could also have been influenced by the fact that the majority of these patients have been included in large interventional treatment studies.

Further, although no new safety signals were identified in this study, the relative infrequency of adverse events makes ongoing evaluation of larger cohorts important.

Future efforts should focus on PRO measures driven and developed by patients undergoing the procedure and applied to prospective patient cohorts.^17^ The continued study of PRO data and development of the current instrument is essential in order to meet the needs of the diverse patient group that will undergo biopsy as the method is implemented in further studies.
