# Supplementary material for: Patient-reported outcomes and safety in patients undergoing synovial biopsy: comparison of ultrasound-guided needle biopsy, ultrasound-guided portal and forceps and arthroscopic-guided synovial biopsy techniques in five centres across Europe
Source: RMD Open. 2018 Oct 26;4(2):e000799. doi: 10.1136/rmdopen-2018-000799 (PMC6241983; doi:10.1136/rmdopen-2018-000799)
Supplement: Supplementary data [file rmdopen-2018-000799supp001.docx]

**Supplementary material**

**Table 1A: Origin of the included procedure data:**

|  | **US-NB** | **US-P&F** | **AGSB** | **Total** | **P-value** |
| --- | --- | --- | --- | --- | --- |
| **n (%)** | 402 (76.7) | 65 (12.4) | 57 (10.9) | 524 (100.0) |  |
| **Study Site** |  |  |  |  |  |
| Barts and the London  (R4RA trial), n (%) | 123 (30.6) | 0 (0.0) | 22 (38.6) | 145 (27.7) |  |
| Barts and the London (STRAP trial), n (%) | 71 (17.7) | 10 (15.4) | 2 (3.5) | 83 (15.8) |  |
| Odense, Denmark, n (%) | 85 (21.1) | 0 (0.0) | 13 (22.8) | 98 (18.7) |  |
| Brussels, Belgium, n (%) | 122 (30.3) | 0 (0.0) | 0 (0.0) | 122 (23.3) |  |
| Birmingham, UK, n (%) | 1 (0.2) | 55 (84.6) | 0 (0.0) | 56 (10.7) |  |
| Lisbon, Portugal, n (%) | 0 (0.0) | 0 (0.0) | 20 (35.1) | 20 (3.8) | 0.00 |

R4RA: Response - Resistance to Rituximab versus Tocilizumab in RA, STRAP: Stratification of Biologic Therapies for RA by Pathobiology

**Table 1B: Origin of the included procedure data from the R4RA and STRAP trials**

| **Centres*** | **n** | **Percent** |
| --- | --- | --- |
| London (UK) | 72 | 31.6 |
| Louvain (BE) | 29 | 12.7 |
| Newcastle (UK) | 13 | 5.70 |
| Cardiff (UK) | 12 | 5.26 |
| Leeds (UK) | 12 | 5.26 |
| Lissabon (PT) | 11 | 4.82 |
| Basildon (UK) | 10 | 4.39 |
| Oxford (UK) | 10 | 4.39 |
| Southampton (UK) | 9 | 3.95 |
| Other centres | 50 | 21.93 |
| Total | 228 | 100 |

*Centres contributing 9 or more procedures to the study is listed.

**Table 2: Detailed Treatment Data**

| **Treatment at time of biopsy** | **US-NB** | **US-P&F** | **AGSB** | **Total** | **P-value*** |
| --- | --- | --- | --- | --- | --- |
| **n (%)** | 402 (76.7) | 65 (12.4) | 57 (10.9) | 524 (100.0) |  |
| **Therapy** |  |  |  |  |  |
| Oral steroid, n (%) | 64 (15.9) | 2 (3.1) | 13 (22.8) | 79 (15.1) | 0.01 |
| Naive, n (%) | 131 (35.2) | 58 (89.2) | 25 (51.0) | 214 (44.0) |  |
| MTX, n (%) | 150 (40.3) | 4 (6.2) | 21 (42.9) | 175 (36.0) |  |
| HQ, n (%) | 4 (1.1) | 0 (0.0) | 0 (0.0) | 4 (0.8) |  |
| SZ, n (%) | 6 (1.6) | 0 (0.0) | 0 (0.0) | 6 (1.2) |  |
| LFL, n (%) | 5 (1.3) | 1 (1.5) | 0 (0.0) | 6 (1.2) |  |
| MTX+HQ, n (%) | 26 (7.0) | 2 (3.1) | 1 (2.0) | 29 (6.0) |  |
| MTX+LFL, n (%) | 3 (0.8) | 0 (0.0) | 0 (0.0) | 3 (0.6) |  |
| LFL+SZ, n (%) | 2 (0.5) | 0 (0.0) | 0 (0.0) | 2 (0.4) |  |
| HPQ+SZ, n (%) | 2 (0.5) | 0 (0.0) | 0 (0.0) | 2 (0.4) |  |
| MTX+HQ+SZ, n (%) | 19 (5.1) | 0 (0.0) | 1 (2.0) | 20 (4.1) |  |
| Other | 1 (0.3) | 0 (0.0) | 0 (0.0) | 1 (0.2) |  |
| Biological treatment alone | 23 (6.2) | 0 (0.0) | 1 (2.0) | 24 (4.9) | <0.01 |
| Biological treatment in combination with DMARD | 30 (7.5) | 1 (1.5) | 0 (0.0) | 31 (5.9) | 0.02 |
| **Biological Therapy** |  |  |  |  |  |
| TNF-alpha inhibitor, n (%) | 33 (62.3) | 1 (100.0) | 1 (100.0) | 35 (63.6) |  |
| Rituximab, n (%) | 8 (15.1) | 0 (0.0) | 0 (0.0) | 8 (14.5) |  |
| Tocilizumab, n (%) | 9 (17.0) | 0 (0.0) | 0 (0.0) | 9 (16.4) |  |
| Orencia, n (%) | 3 (5.7) | 0 (0.0) | 0 (0.0) | 3 (5.5) | 0.98 |

* P value by Chi-square Test; MTX: Methotrexate; HQ: Hydroxychloroquine; SZ: Sulfasalazine; LFL: Leflunomide; DMARD: Disease Modifying Anti Rheumatic Drugs; TNF-alpha inhibitor: Tumor Necrosis Factor-alpha inhibitor

**Table 3: Comparison of PRO data between the methods**

|  | **US-NB** | **US-P&F** | **AGSB** | **Total** | **P-value** | **P-value*** | **Missing** |
| --- | --- | --- | --- | --- | --- | --- | --- |
| **Biopsy procedures, n (%)** | **402 (76.7)** | **65 (12.4)** | **57 (10.9)** | **524 (100.0)** |  |  |  |
| **Pain, Swelling and stiffness before and after biopsy** | | |  |  |  |  |  |
| Pre Pain, mean (SD) | 52.21 (29.14) | 45.60 (32.90) | 62.44 (23.98) | 52.51 (29.36) | <0.01 | <0.01 | 2.5 |
| Post Pain, mean (SD) | 39.47 (29.94) | 32.46 (27.42) | 43.16 (22.62) | 38.91 (29.03) | 0.09 | 0.19 | 7.2 |
| Pre Swelling, mean (SD) | 50.29 (30.70) | 41.02 (33.11) | 53.09 (29.26) | 49.40 (30.99) | 0.09 | <0.01 | 5.3 |
| Post Swelling, mean (SD) | 35.95 (30.47) | 31.18 (26.66) | 36.46 (26.00) | 35.35 (29.52) | 0.46 | 0.17 | 9.5 |
| Pre Stiffness, mean (SD) | 51.48 (30.32) | 47.28 (33.89) | 48.79 (30.13) | 50.62 (30.77) | 0.59 | 0.03 | 5.3 |
| Post Stiffness, mean (SD) | 37.72 (30.45) | 33.75 (28.01) | 36.14 (24.91) | 37.01 (29.57) | 0.61 | 0.18 | 9.3 |
| **Difference between Post and Pre values** | |  |  |  |  |  |  |
| Delta Pain, mean (SD) | -12.90 (27.74) | -13.14 (30.36) | -20.94 (28.91) | -13.76 (28.27) | 0.16 | 0.19 | 7.6 |
| Delta Swelling, mean (SD) | -14.67 (29.57) | -9.83 (27.74) | -18.68 (35.03) | -14.43 (29.96) | 0.30 | 0.17 | 9.9 |
| Delta Stiffness, mean (SD) | -13.70 (28.92) | -13.52 (32.25) | -14.18 (37.49) | -13.72 (30.32) | 0.99 | 0.18 | 9.7 |

*P-value adjusted for biopsy method, disease activity, intra-articular or intramuscular corticosteroid injection during biopsy. Further, Post and delta values were also adjusted for pre-value.
